# Supplementary material for: Clinicopathologic and Prognostic Association of GRP94 Expression in Colorectal Cancer with Synchronous and Metachronous Metastases
Source: Int J Mol Sci. 2021 Jun 30;22(13):7042. doi: 10.3390/ijms22137042 (PMC8267630; doi:10.3390/ijms22137042)
Supplement: Supplementary file 1 [file ijms-22-07042-s001.zip › Table S1.pdf]

Table S1. Baseline clinicopathologic characteristics of advanced colorectal cancer with distant metastasis.

Factors	No	GRP94		
		GRP94 (−)	GRP94 (+)	*P*
Age				0.230
<65	119	46 (38.7%)	73 (61.3%)	
≥65	70	21 (30.0%)	49 (70.0%)	
Sex				0.510
M	102	34 (33.3%)	68 (66.7%)	
F	87	33 (37.9%)	54 (62.1%)	
Location				0.292
Right sided	48	14 (29.2%)	34 (70.8%)	
Left sided	141	53 (37.6%)	88 (62.4%)	
Differentiation				0.159
Low grade	164	55 (33.5%)	109 (66.5%)	
High grade	25	12 (48.0%)	13 (52.0%)	
Lymphatic invasion				0.588
Absent	64	21 (32.8%)	43 (67.2%)	
Present	125	46 (36.8%)	79 (63.2%)	
Venous invasion				0.635
Absent	131	45 (34.4%)	86 (65.6%)	
Present	58	22 (37.9%)	36 (62.1%)	
Perineural invasion				0.623
Absent	92	31 (33.7%)	61 (66.3%)	
Present	97	36 (37.1%)	61 (62.9%)	
pT stage				0.557
pT2-3	116	43 (37.1%)	73 (62.9%)	
pT4	73	24 (32.9%)	49 (67.1%)	
pN stage				0.927
pN0	36	13 (36.1%)	23 (63.9%)	
pN+	153	54 (35.3%)	99 (64.7%)	
MSI				0.553
MSS/MSI-low	186	67 (36.0%)	119 (64.0%)	
MSI-high	3	0 (0%)	3 (100%)	
EGFR				0.110
Negative	116	36 (31.0%)	80 (69.0%)	
Positive	73	31 (42.5%)	42 (57.5%)	
KRAS mutation				0.952
Wild type	88	31 (35.2%)	57 (64.8%)	

Mutant	101	36 (35.6%)	65 (64.4%)	
PIK3CA mutation				0.021
Wild type	163	63 (38.7%)	100 (61.3%)	
Mutant	26	4 (15.4%)	22 (84.6%)	
BRAF mutation				1.000
Wild type	182	65 (35.7%)	117 (64.3%)	
Mutant	7	2 (28.6%)	5 (71.4%)	
HER2 amplification (n = 185)				0.164
Negative	176	64 (36.4%)	112 (63.6%)	
Positive	9	1 (11.1%)	8 (88.9%)	
