# Supplementary material for: Clinicopathologic and Prognostic Association of GRP94 Expression in Colorectal Cancer with Synchronous and Metachronous Metastases
Source: Int J Mol Sci. 2021 Jun 30;22(13):7042. doi: 10.3390/ijms22137042 (PMC8267630; doi:10.3390/ijms22137042)
Supplement: Supplementary file 1 [file ijms-22-07042-s001.zip › Table S2.pdf]

Table S2. GRP94 expression status in the primary tumor, its invasive border, and the corresponding metastasis of CRC.

Primary tumor	Invasive border		*P**	Metastasis		*P**
	GRP94 (-)	GRP94 (+)		GRP94 (-)	GRP94 (+)	
Total			0.311			0.012
GRP94 (-)	46 (76.7%)	14 (23.3%)		40 (62.5%)	24 (37.5%)	
GRP94 (+)	21 (18.3%)	94 (81.7%)		46 (43.4%)	60 (56.6%)	
SM subgroup			0.078			0.003
GRP94 (-)	32 (84.2%)	6 (15.8%)		26 (70.3%)	11 (29.7%)	
GRP94 (+)	15 (19.0%)	64 (81.0%)		31 (43.7%)	40 (56.3%)	
MM subgroup			0.791			0.851
GRP94 (-)	14 (63.6%)	8 (36.4%)		14 (51.9%)	13 (48.1%)	
GRP94 (+)	6 (16.7%)	30 (83.3%)		15 (42.9%)	20 (57.1%)	

*McNemar's paired test
