# Supplementary material for: Clinicopathologic and Prognostic Association of GRP94 Expression in Colorectal Cancer with Synchronous and Metachronous Metastases
Source: Int J Mol Sci. 2021 Jun 30;22(13):7042. doi: 10.3390/ijms22137042 (PMC8267630; doi:10.3390/ijms22137042)
Supplement: Supplementary file 1 [file ijms-22-07042-s001.zip › Table S3.pdf]

Table S3. GRP94 expression at different metastatic sites.

Location of metastasis	GRP94 expression		*P*
	Negative	Positive	
Total			0.004
Liver	34 (38.6%)	54 (61.4%)	
Lung	10 (24.4%)	31 (75.6%)	
Peritoneal seeding	9 (24.3%)	28 (75.7%)	
Ovary	11 (61.1%)	7 (38.9%)	
Distant node	3 (60.0%)	2 (40.0%)	
SM subgroup			0.044
Liver	23 (35.9%)	41 (64.1%)	
Lung	2 (18.2%)	9 (81.8%)	
Peritoneal seeding	6 (19.4%)	25 (80.6%)	
Ovary	7 (53.8%)	6 (46.2%)	
Distant node	2 (50.0%)	2 (50.0%)	
MM subgroup			0.066
Liver	11 (45.8%)	13 (54.2%)	
Lung	8 (26.7%)	22 (73.3%)	
Peritoneal seeding	3 (50.0%)	3 (50.0%)	
Ovary	4 (80.0%)	1 (20.0%)	
Distant node	1 (100%)	0 (0%)	
