# Supplementary material for: Clinicopathologic and Prognostic Association of GRP94 Expression in Colorectal Cancer with Synchronous and Metachronous Metastases
Source: Int J Mol Sci. 2021 Jun 30;22(13):7042. doi: 10.3390/ijms22137042 (PMC8267630; doi:10.3390/ijms22137042)
Supplement: Supplementary file 1 [file ijms-22-07042-s001.zip › Table S4.pdf]

Table S4. Univariate analysis for overall survival in CRC patients by synchronous and metachronous metastases

Factor	Hazard ratio (95% CI)	Significance
Total		
Age (≥65 vs <65)	1.588 (1.024-2.462)	0.039
Sex (F vs M)	1.363 (0.880-2.110)	0.165
Location (left sided vs right sided)	0.596 (0.373-0.952)	0.030
Differentiation (high grade vs low grade)	1.821 (1.022-3.244)	0.042
Lymphatic invasion (present vs absent)	2.730 (1.557-4.787)	<0.001
Vascular invasion (present vs absent)	1.173 (0.731-1.882)	0.509
Neural invasion (present vs absent)	2.398 (1.506-3.817)	<0.001
pT stage (pT4 vs pT2-3)	2.154 (1.388-3.343)	0.001
pN stage (pN+ vs pN0)	3.832 (1.754-8.374)	0.001
MSI (MSI-high vs MSS/MSI-low)	0.426 (0-84.288)	0.048
EGFR (present vs negative)	1.414 (0.912-2.191)	0.121
KRAS (mutant vs wild type)	0.990 (0.640-1.532)	0.964
PIK3CA (mutant vs wild type)	0.798 (0.411-1.548)	0.505
BRAF (mutant vs wild type)	1.868 (0.683-5.110)	0.224
HER2 (amplification vs no amplification)	1.527 (0.612-3.806)	0.364
CD3+ TILs (low vs high)	0.731 (0.471-1.135)	0.163
CD4+ TILs (low vs high)	0.737 (0.407-1.335)	0.314
CD8+ TILs (low vs high)	0.797 (0.458-10387)	0.422
Foxp3+ TILs (low vs high)	0.049 (0-74001.982)	0.696
GRP94 (positive vs negative)	0.821 (0.526-1.281)	0.385
Synchronous metastasis group		
Age (≥65 vs <65)	1.945 (1.176-3.219)	0.010
Sex (F vs M)	0.807 (0.494-1.320)	0.394
Location (left sided vs right sided)	0.682 (0.409-1.137)	0.142
Differentiation (high grade vs low grade)	1.500 (0.761-2.957)	0.241
Lymphatic invasion (present vs absent)	2.073 (1.102-3.899)	0.024
Vascular invasion (present vs absent)	0.839 (0.494-1.423)	0.514
Neural invasion (present vs absent)	1.553 (0.934-2.582)	0.089
pT stage (pT4 vs pT2-3)	1.410 (0.861-2.309)	0.172
pN stage (pN+ vs pN0)	1.452 (0.582-3.624)	0.424
MSI (MSI-high vs MSS/MSI-low)	0.049 (0-76976.995)	0.679
EGFR (present vs negative)	1.327 (0.807-2.183)	0.265
KRAS (mutant vs wild type)	1.190 (0.726-1.952)	0.490
PIK3CA (mutant vs wild type)	0.780 (0.385-1.581)	0.491
BRAF (mutant vs wild type)	0.980 (0.306-3.133)	0.972
HER2 (amplification vs no amplification)	1.061 (0.422-2.667)	0.900
CD3+ TILs (low vs high)	0.790 (0.481-1.297)	0.352
CD4+ TILs (low vs high)	1.061 (0.566-1.991)	0.853
CD8+ TILs (low vs high)	0.897 (0.466-1.726)	0.744

GRP94 (positive vs negative)	0.577 (0.349-0.953)	0.032
Metachronous metastasis group		
Age (≥65 vs <65)	1.970 (0.749-5.182)	0.169
Sex (F vs M)	6.363 (1.377-9.600)	0.009
Location (left sided vs right sided)	0.799 (0.226-2.822)	0.728
Differentiation (high grade vs low grade)	3.032 (0.983-9.353)	0.054
Lymphatic invasion (present vs absent)	3.992 (1.146-13.911)	0.030
Vascular invasion (present vs absent)	2.000 (0.699-5.722)	0.196
Neural invasion (present vs absent)	8.082 (2.307-28.319)	0.001
pT stage (pT4 vs pT2-3)	3.083 (1.132-8.394)	0.028
pN stage (pN+ vs pN0)	63679 (1.505-29.652)	0.013
MSI (MSI-high vs MSS/MSI-low)	0.628 (0-10696-985)	0.628
EGFR (present vs negative)	1.836 (0.708-4.761)	0.212
KRAS (mutant vs wild type)	0.606 (0.229-1.601)	0.312
PIK3CA (mutant vs wild type)	0.427 (0.057-3.225)	0.410
BRAF (mutant vs wild type)	21.162 (2.201-203.453)	0.008
CD3+ TILs (low vs high)	0.771 (0.295-2.017)	0.596
CD4+ TILs (low vs high)	0.202 (0.027-1.524)	0.121
CD8+ TILs (low vs high)	0.899 (0.311-2.598)	0.844
Foxp3+ TILs (low vs high)	0.049 (0-1377473099)	0.822
GRP94 (positive vs negative)	1.208 (0.458-3.185)	0.703
