# Supplementary figures and images for: Clinicopathologic and Prognostic Association of GRP94 Expression in Colorectal Cancer with Synchronous and Metachronous Metastases
Source: Int J Mol Sci. 2021 Jun 30;22(13):7042. doi: 10.3390/ijms22137042 (PMC8267630; doi:10.3390/ijms22137042)

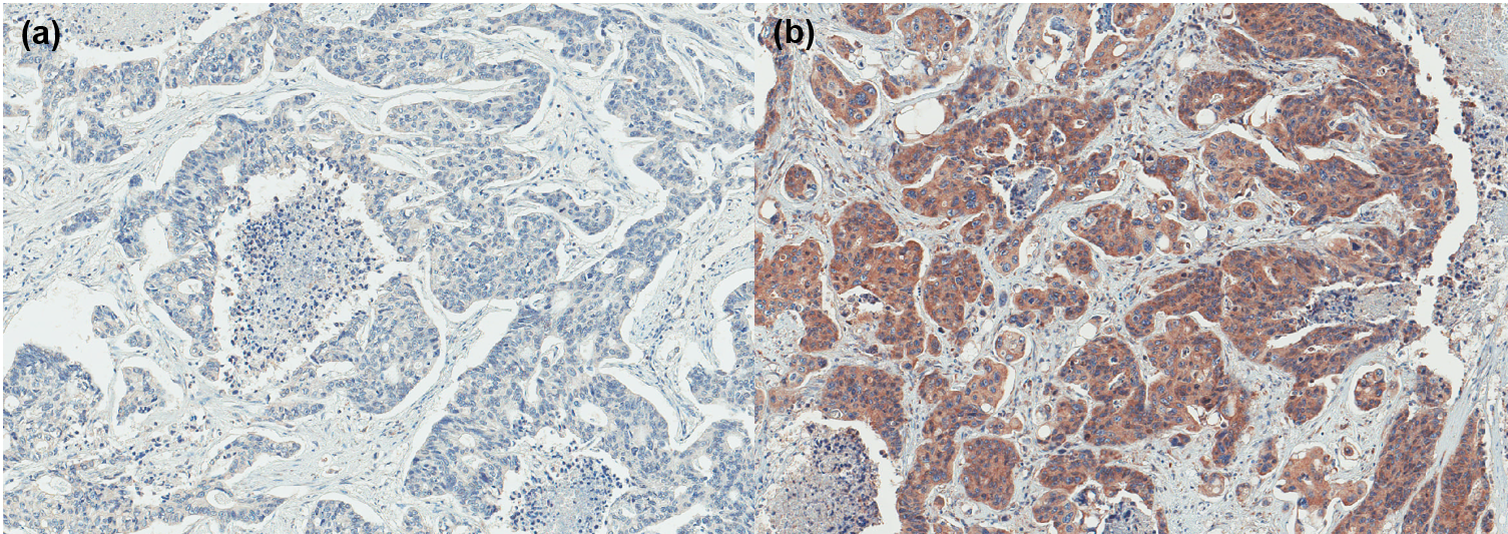

Supplement: Supplementary file 1 [file ijms-22-07042-s001.zip › Figure S1.tif]

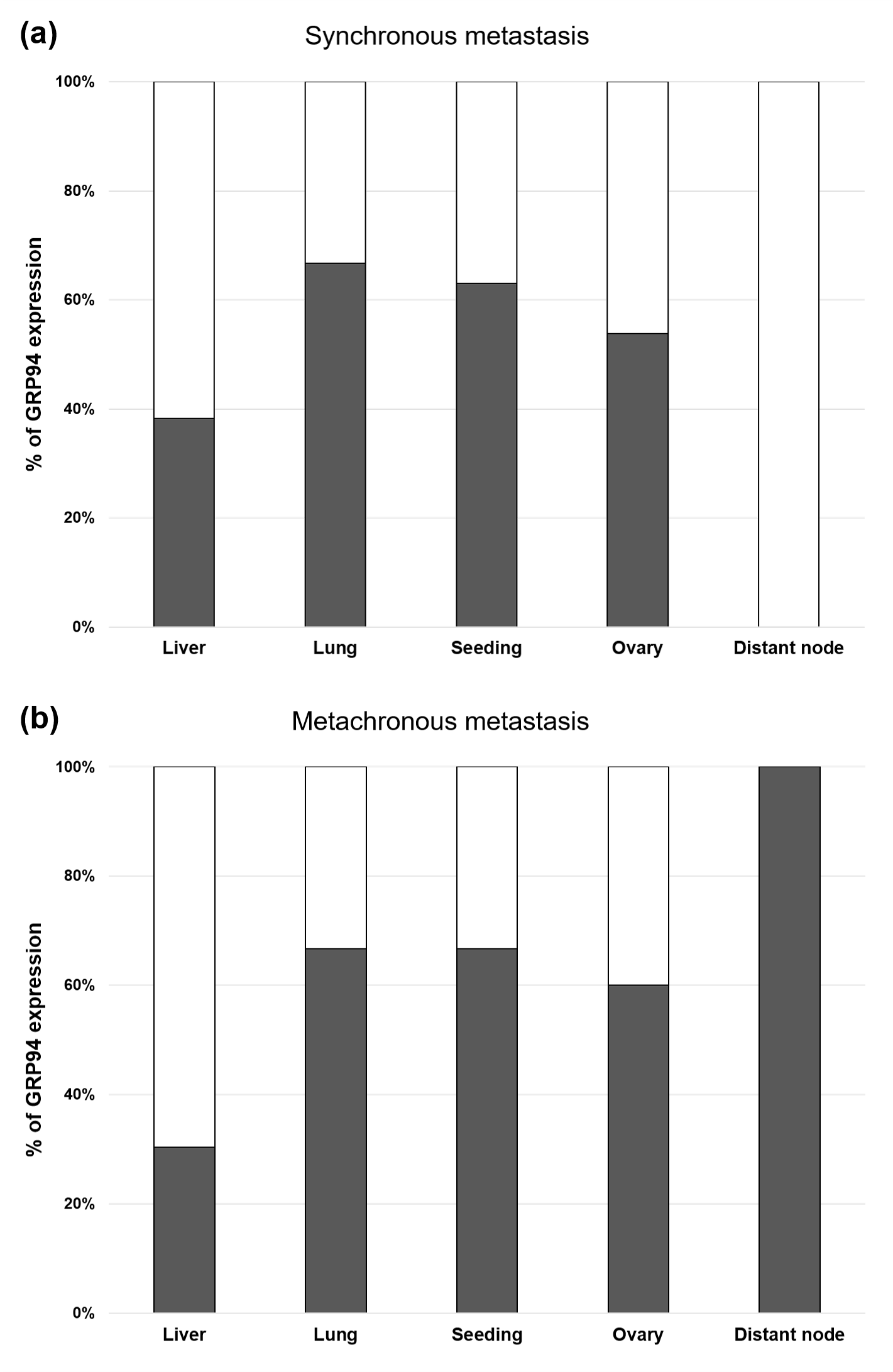

Supplement: Supplementary file 1 [file ijms-22-07042-s001.zip › Figure S2.tif]
